# Blood lipid levels and all-cause mortality in older adults: the Chinese Longitudinal Healthy Longevity Survey 2008-2018

**DOI:** 10.4178/epih.e2022054

**Published:** 2022-07-05

**Authors:** Rongxi Wang, Xiaoyue Yu, Zhiqiang Wang, Yujie Liu, Hui Chen, Shangbin Liu, Chen Xu, Yingjie Chen, Xin Ge, Danni Xia, Ruijie Chang, Gang Xu, Mi Xiang, Ying Wang, Tian Shen, Fan Hu, Yong Cai

**Affiliations:** School of Public Health, Shanghai Jiao Tong University School of Medicine, Shanghai, China

**Keywords:** Blood lipids, All-cause mortality, Elderly

## Abstract

**OBJECTIVES:**

Proper blood lipid levels are essential for survival in older adults, but inconsistent relationships have been reported between blood lipids and all-cause mortality in the elderly.

**METHODS:**

This retrospective longitudinal study analyzed data from 1,067 Chinese older adults enrolled in the Chinese Longitudinal Healthy Longevity Survey collected in 2008 and followed up until death or December 31, 2018. The outcome was all-cause mortality. Multivariate Cox regression analyses were used to estimate hazard ratios (HRs) and 95% confidence intervals (CIs) with stratification by age (60-80, 80-100, or ≥100 years) for further analysis. The survival probability according to lipid profile quartiles was calculated using Kaplan-Meier curves and the log-rank test.

**RESULTS:**

The participants’ mean age was 84.84 years, and 57.0% were female. In total, 578 individuals died, and 277 were lost to follow-up. The mean total cholesterol (TC), high-density lipoprotein cholesterol (HDL-C), and low-density lipoprotein cholesterol (LDL-C) levels were higher among those who died than among those who survived. Participants in the second HDL-C quartile and the highest LDL-C and triglyceride (TG) quartiles had 28% higher, 23% lower, and 49% lower risks of all-cause mortality, respectively. After further adjustment, the associations remained except for HDL-C, and additional associations were observed between all-cause mortality and the third TC and LDL-C quartiles and the second TG quartile (HR, 1.44; 95% CI, 1.01 to 2.06; HR, 0.68; 95% CI, 0.49 to 0.94; HR, 0.79; 95% CI, 0.62 to 0.99, respectively).

**CONCLUSIONS:**

Older adults should maintain an LDL-C level of 1.91-2.47 mmol/L and a TG level of no less than 1.66 mmol/L.

## INTRODUCTION

A consensus has been reached on the importance of maintaining low blood lipid levels to prevent cardiovascular disease due to the well-documented association between lipid levels and the risk of cardiovascular disease and death in middle-aged people. The survival advantage related to lower blood lipids may differ, however, for the elderly population. The U-shaped or inverse relationships found between total cholesterol (TC) levels and all-cause mortality in older people in Eastern and Western countries indicated that lower TC corresponded to a higher risk of death [1-5]. Several studies found low blood lipid levels to be associated with frailty and infectious diseases, and others identified an association with higher cancer mortality rates [6,7]. Furthermore, several studies have analyzed the proportions of different fractions of cholesterol such as low-density lipoprotein cholesterol (LDL-C), high-density lipoprotein cholesterol (HDL-C), and triglycerides (TG). Coronary heart disease (CHD) mortality has been found to have a positive relationship with LDL-C and TG and a negative relationship with HDL-C. However, studies on the relationship between all-cause mortality and lipid profiles have yielded inconsistent results, especially concerning the older population [8-13]. The general pattern that has emerged from prospective studies is that TC and LDL-C have a direct relationship with CHD mortality and an inverse association with all-cause mortality and mortality related to other chronic diseases, whereas HDL-C protects against both cardiovascular and non-cardiovascular disease mortality. However, whether this pattern corresponds to the elderly population in China remains unclear.

In this study, baseline characteristics collected in 2008 and plasma biochemical parameters measured in 2009 from the fifth follow-up survey and survival information from the following 10 years from the Chinese Longitudinal Healthy Longevity Survey (CLHLS) were statistically analyzed to explore the associations between blood lipid fractions and all-cause mortality in elderly people in China and determine a reference lipid level for the improved survival of the elderly.

## MATERIALS AND METHODS

### Study population

The study population was selected from the CLHLS collected in 2008/2009 and 2018. The CLHLS is the first and largest nationwide, community-based, longitudinal prospective survey in China [14]. The CLHLS was designed to identify the determinants of the health and longevity of older adults. The CLHLS is conducted by randomly selecting participants from counties and cities across 22 provinces in China, covering about 85% of the total population of China [15]. The CLHLS was started in 1998, and follow-up surveys were conducted in 2000, 2002, 2005, 2008/2009, 2011/2012, 2014, and 2017/2018 [16]. The CLHLS contains a wide variety of information on demographics, lifestyle, diet, health status, and the daily activities of older people. In 2009, a biomarker sub-study was launched in 8 regions, including Sanshui in Guangdong Province, Yongfu in Guangxi Autonomous Area, Chengmai in Hainan Province, Xiayi in Henan Province, Zhongxiang in Hubei Province, Mayang in Hunan Province, Laizhou in Shandong Province, and Rudong in Jiangsu Province.

**Figure 1.**
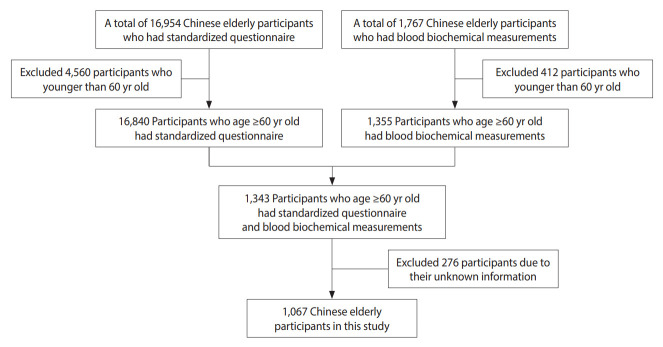
Flow chart on the selection of study population.

We included 1,067 Chinese elderly participants in this study after applying the following exclusion criteria: (1) participants younger than 60 years of age and (2) participants with missing information. The flow chart of the study population is shown in Figure 1.

### Data collection

We collected information using a standardized questionnaire administered through household interviews. The information included demographic data such as age, sex, residence, marital status, and economic status. After the household interview, each participant was asked to undergo anthropometric measurements, which included systolic blood pressure (SBP, mmHg), diastolic blood pressure (DBP, mmHg), and body mass index (BMI, kg/m^2^). Blood biochemistry tests were analyzed in this study, including blood urea nitrogen (mmol/L), plasma creatine (mmol/L), uric acid (μmol/L), plasma glucose (mmol/L), TC (mmol/L), HDL-C (mmol/L), LDL-C (mmol/L), and TG (mmol/L) levels.

### Biochemical measurements

Fasting venous blood samples were collected by trained medical personnel from all willing participants after fasting overnight. Five milliliters of fasting venous blood were collected in heparin anticoagulant vacuum tubes and centrifuged at 20°C and 2,500 RPM for 10 minutes. The plasma was isolated and frozen at -20°C, shipped through a cold chain to the central laboratory at Capital Medical University in Beijing, and stored at -80°C until biochemical analysis. Blood urea nitrogen, plasma creatine, uric acid, plasma glucose, TC, HDL-C, LDL-C, and TG levels were measured using an automatic biochemical analyzer (Hitachi 7180; Hitachi High-Technologies, Tokyo, Japan).

### Study outcome during prospective follow-up

The outcome of our study was all-cause mortality from January 1, 2008, to December 31, 2018. Mortality status was obtained from the publicly available CLHLS dataset, which contained data on the vital status of the survey participants from baseline to December 31, 2018. If a participant missed the follow-up visit, the survival time was defined as the interval between baseline and the time of the missed visit.

### Statistical analysis

The study population was divided into 4 groups based on quartiles of lipid profiles. Continuous variables were presented as the mean and standard deviation (mean± SD) and compared using one -way analysis of variance. The categorical variables were presented in terms of numerical counts (%) and were compared using the chi-square test. Multivariate Cox regression analyses were used to investigate the relationships between quartiles of lipid profiles with all-cause mortality, and the results were presented as hazard ratios (HRs) and 95% confidence intervals (CIs). Associations between quartiles of lipid profiles concerning all-cause mortality were assessed using 4 models as follows: quartiles for TC, age, sex, category of residence, marital status, and economic status were entered in model 1; model 2 was further adjusted for SBP, DBP, and BMI; model 3 was further adjusted for blood urea nitrogen, plasma creatine, uric acid, and plasma glucose levels; and quartiles for HDL-C, LDL-C, and TG levels were entered in model 4. A similar process was applied to quartiles of HDL-C, LDL-C, and TG. Age (60-80, 80-100, ≥ 100 years), sex (male, female), area of residence (city/town, rural), marital status (unmarried, married), economic status (< 10,000, ≥ 10,000 renminbi/yr), smoking status (no, yes) and drinking status (no, yes) were adjusted as categorical variables while SBP, DBP, BMI, blood urea nitrogen, plasma creatine, uric acid, and plasma glucose levels were adjusted as continuous variables. To confirm associations, sensitivity analysis was performed based on receiver operating characteristic (ROC) curves and the corresponding area under the curve (AUC). In addition, we performed subgroup analyses of the association between lipid profiles and all-cause mortality by age group (60-80, 80-100, or ≥ 100 years). Furthermore, the survival probability according to each lipid profile quartile was calculated using Kaplan-Meier curves, and the log-rank test was performed to analyze differences between quartiles. Statistical analysis was performed using the R version 4.03 (R Core Team, Vienna, Austria). A 2-sided p-value of < 0.05 was considered to indicate statistical significance.

### Ethics statement

The CLHLS study was conducted according to the guidelines of the Declaration of Helsinki and approved by the Research Ethics Committee of Peking University (IRB00001052-13074). All participants provided written informed consent.

## RESULTS

The sample consisted of 1,067 individuals aged 65-107 years old (mean±SD, 84.84±13.15), and the majority of participants were female (57.0%). The mean follow-up time was 62.97 months (range, 2-120). A total of 578 deaths (54.2%) were recorded, and 277 participants (26.0%) were lost to follow-up, the majority of whom were females. The mean TC, HDL-C, and LDL-C levels were higher in those who died than for those who survived, while TG levels were not (Table 1). The baseline characteristics of the study population according to the TC quartile are shown in Supplementary Material 1. The participants in the highest TC quartile were more likely to be between 80 years and 100 years of age, female, non-smokers, and non-drinkers as well as have a lower economic status, have higher levels of HDL-C and LDL-C, and reside in a rural area. Participants with the lowest concentrations of TC were more likely to have higher levels of plasma creatine and TG. The baseline characteristics according to blood lipid fraction quartile, including HDL-C, LDL-C, and TG, are shown in Supplementary Materials 1-4. Age-specific and sex-specific baseline characteristics are shown in Supplementary Material 5.

### All-cause mortality risk and lipid levels in different adjusted models

As shown in Table 2, after adjustment for socio-demographic and lifestyle characteristics, participants in the second HDL-C quartile and the highest TG quartile had a 32% higher risk and 59% lower risk of all-cause mortality, respectively (HR, 1.32; 95% CI, 1.03 to 1.68; HR, 0.59; 95% CI, 0.46 to 0.77), compared to those in the lowest HDL-C and TG quartiles in model 1. After further adjustment in model 2 (based on model 1 with adjustments for SBP, DBP, and BMI), model 3 (based on model 2 with adjustments for blood urea nitrogen, plasma creatine, uric acid, and plasma glucose), and model 4 (based on model 3 with adjustments for TC, HDL-C, LDL-C, and TG), the association between TG and all-cause mortality remained significant in addition to the association between HDL-C and all-cause mortality other than that in model 4. In the fully adjusted model (model 4), however, an extra association between all-cause mortality and participants in the third TC and LDL-C quartiles and the second TG quartile appeared in comparison to other models (HR, 1.44; 95% CI, 1.01 to 2.06; HR, 0.68; 95% CI, 0.49 to 0.94; HR, 0.79; 95% CI, 0.62 to 0.99). Furthermore, the third LDL-C quartile protected against all-cause mortality in model 3 only (HR, 0.77; 95% CI, 0.61 to 0.99), and the association between LDL-C and all-cause mortality was not significant in the other models.

### Sensitivity analysis

**Figure 2.**
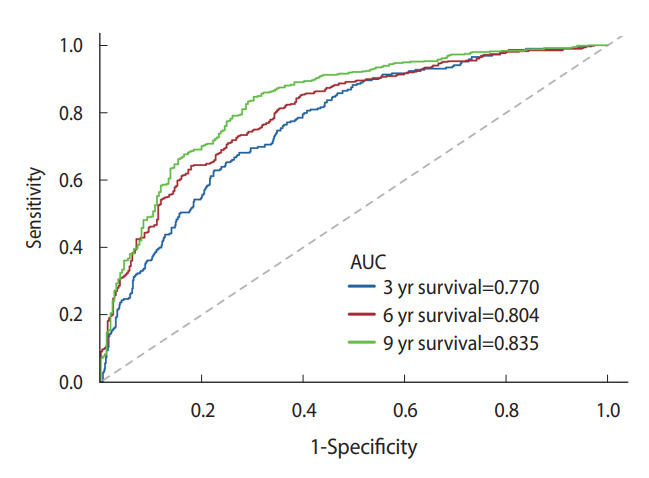
ROC curves of the multivariate model at the 3-, 6-, and 9-year points all-cause mortality. ROC, receiver operating characteristic; AUC, area under the curve.

The multivariate model’s discriminative performance was assessed using ROC curves and the AUC. The AUCs for 3-year, 6-year, and 9-year all-cause mortality were 0.770, 0.804, and 0.835, respectively (Figure 2).

### Subgroup analysis

Further exploration of the associations between TC (and its fractions) and all-cause mortality based on 3 different age groups is shown in Supplementary Materials 6-8. For participants between 60 years and 80 years of age, an increased risk of all-cause mortality was observed in all models for the second HDL-C quartile only. For participants between 80 years and 100 years of age, there was an increased risk of all-cause mortality for those in the third TC quartile, but a protective effect against all-cause mortality was observed in the highest TG quartile in all models. In addition, those in the second TC quartile had an increased risk of all-cause mortality (HR, 1.62; 95% CI, 1.08 to 2.43), while the second LDL-C quartile showed a protective effect against all-cause mortality in model 4 only (HR, 0.67; 95% CI, 0.47 to 0.95). For those over 100 years of age, none of the associations between TC quartiles (and the fractions of cholesterol) and all-cause mortality were statistically significant in any of the models.

### Survival analysis

**Figure 3.**
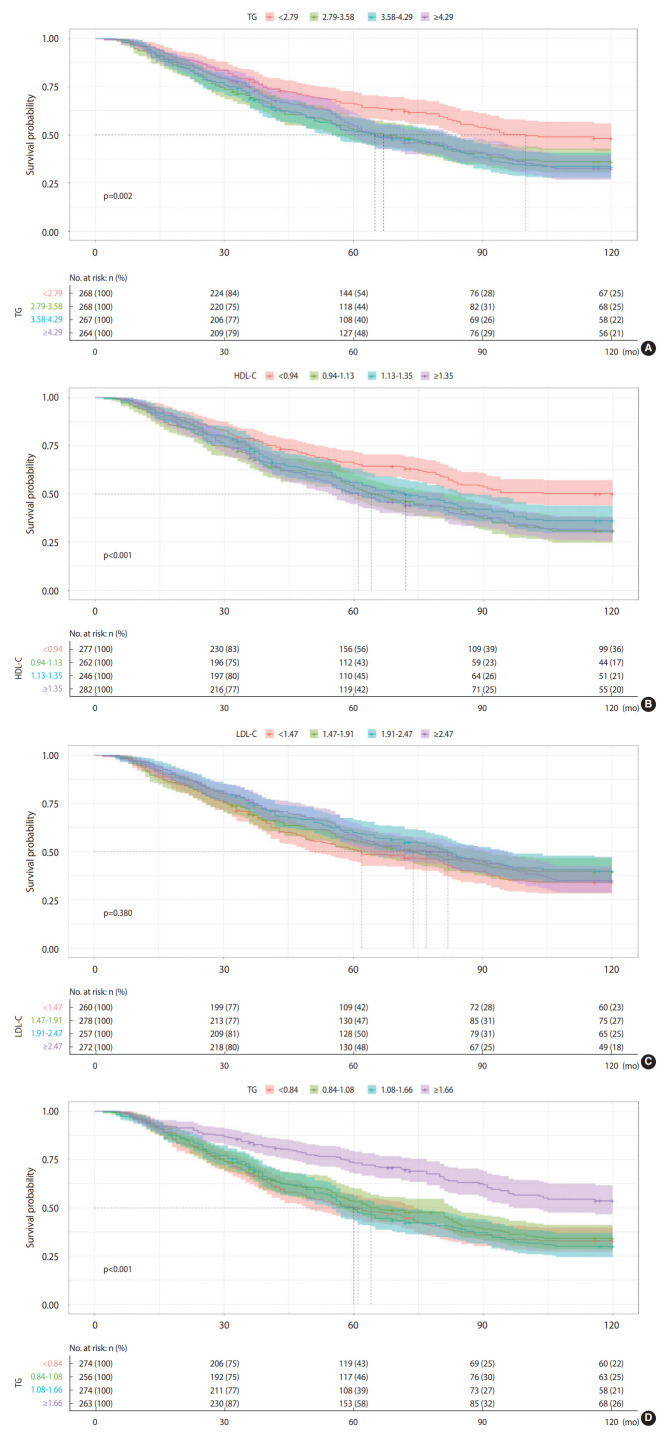
Kaplan–Meier survival curve estimates for all-cause mortality according to the (A) total cholesterol (TC), (B) high-density lipoprotein cholesterol (HDL-C), (C) low-density lipoprotein cholesterol (LDL-C), and (D) triglycerides (TG). in the study population.

Figure 3 shows the Kaplan-Meier survival curves for all-cause mortality in 4 different blood lipid groups (TC, HDL-C, LDL-C, and TG). For the relationship between TC, HDL-C, and all-cause mortality, participants in the lowest quartiles were more likely to have a significantly higher survival probability (p=0.002, < 0.001, respectively) than those in the higher quartiles, while participants in the highest TG quartile were more likely to have a significantly higher survival probability (p<0.001). The survival probability between participants in each of the 4 LDL-C quartiles, however, did not significantly differ (p=0.380).

## DISCUSSION

The present study found that higher LDL-C and TG levels were significantly associated with lower all-cause mortality, indicating better survival, while higher TC levels increased the risk of all-cause mortality and higher HDL-C levels did not increase the subsequent risk of mortality. The mortality risk of older adults decreased by 32% among those in the third LDL-C quartile (1.91-2.47 mmol/L) and by 45% among those in the fourth TG quartile (≥ 1.66 mmol/L). In addition, this trend was inconsistent with regard to each age group. Blood lipid levels may not affect the survival of those aged 100 years and older as much as it does those younger than 100 years of age.

In the model that initially included quartiles of blood lipids, TC, HDL-C, and TG were meaningful. The second and third TC quartiles and second HDL-C quartile increased the risk of death, while the fourth TG quartile decreased all-cause mortality. This echoes the results of past studies [6,8,12,17]. After adjusting for age, sex, area of residence, smoking status, and drinking status, however, the association between HDL-C and all-cause mortality lost its statistical significance. To better determine the relationship between blood lipids and all-cause mortality, the model was further adjusted for BMI, SBP, DBP, blood urea nitrogen, plasma creatine, uric acid, and plasma glucose levels, and higher LDL-C and TG concentrations were found to be associated with lower all-cause mortality, which suggests that high LDL-C and TG levels may be independent protective factors related to all-cause mortality. The lowest mortality risk appeared in the third LDL-C (1.91-2.47 mmol/L) and fourth TG (≥ 1.66 mmol/L) quartiles. Several studies have found low LDL-C and TG levels to be associated with an increased mortality risk [6,12,18,19]. The possible mechanism for these relationships may be the protective role of lipids and lipoproteins in modulating inflammation markers, such as cytokines, C-reactive protein, and oxidized LDL-C [17,20,21]. Low-serum LDL-C increases the risk of infection and sepsis, leading to higher levels of oxidized LDL-C and increased inflammation. Furthermore, LDL-C functions as a carrier of the exogenous coenzyme Q10 which reduces the negative effects of septic shock by acting as an effective radical scavenger [22]. Relationships between low-serum LDL-C levels and the risk of fever, sepsis, and malignancy have been observed in studies of various populations [23-25]. However, the inverse relationship between LDL-C and TG levels and mortality may have been distorted by survival bias, meaning that the individuals most susceptible to the detrimental effects of high-serum cholesterol likely die before reaching a certain age and those who live longer (two-thirds of the study population aged 80 years and older) likely have a beneficial genetic risk profile related to LDL-C concerning familial longevity [2,26]. In addition, blood lipid concentration was found to be sensitive to dietary patterns, especially TG levels, which increase higher intake of dietary carbohydrates [27,28]. Therefore, older adults’ diets may be another potential contributing factor. The positive correlation between TC and all-cause mortality suggests this possibility. There are 2 possible explanations for this positive relationship. First, TC was identified as a risk factor for CHD, which remains a leading cause of death among older adults. Second, this study found that high levels of HDL-C increase the risk of mortality, although not significantly. Past studies have recommended an optimal range of HDL-C concentration related to all-cause mortality and have suggested that excessively high HDL-C levels may negatively affect the survival of older adults [8,29]. This negative effect may be stronger than the protective effect of normal HDL-C levels [29]. The sensitivity analysis revealed that model 4 had excellent discrimination ability, confirming this association.

Blood lipid levels decline from middle-age to old-age [30,31]. Unlike Schupf et al. [6], who attributed the age difference in the relationship between LDL-C and mortality among older adults without dementia to chance, we assumed that the discrepancies in the results of past studies may be partly due to differences in the age limits of the older adults included in study populations [3,11,32,33]. In the current study, we further explored the relationship between blood lipids and all-cause mortality in different age groups. No significant relationships between TC, LDL-C, and TG and all-cause mortality were observed in the 60-80 years age group. Cabrera et al. [11] reported similar findings among Brazilian older adults aged 60-85 years. These results suggest that those in this age range may benefit from monitoring and controlling their lipid levels according to National Cholesterol Education Program guidelines to prevent cardiovascular disease. For those aged 80-100 years old, LDL-C and TG levels played a protective role against mortality and enhanced survival, indicating the importance of maintaining proper LDL-C levels (1.47-1.91 mmol/L) and high TG levels to live longer lives. The survival status of older adults aged 100 years and older did not seem to be affected by blood lipid levels and may depend to a large extent on their genetic profile [34,35].

This study has several important limitations. First, due to constraints in human and material resources, we could not obtain biochemical information of older adults from all 23 regions. The final sample size and representativeness of the study population were not good enough to generalize the results of the study to the national elderly population. Second, it is very difficult to follow-up with elderly participants for 10 years, and the rate of loss to follow-up was relatively high (up to 26.0%) in this study. Third, although the model adjusted for blood pressure, BMI, and other factors, adjustments for several indicators that may also affect the survival status of older people, such as albumin (an indicator of frailty) and diet, were not included in the last model. Fourth, a relationship between blood lipid levels and all-cause mortality does not necessarily mean that blood lipids directly affect survival and death. We did not consider basic diseases, chronic diseases, or exposure to statins among the study population. Blood lipids may affect the course of a disease and ultimately affect survival and death or vice versa [36]. In addition, the cohort in this study was surveyed in the CLHLS and had a low prevalence of underlying diseases.

In conclusion, monitoring blood lipid levels is important for a long and healthy life. Older people should be careful to maintain an LDL-C level of 1.91-2.47 mmol/L, and a TG level of 1.66 mmol/L may benefit the aging process. Future research on older adults is needed to understand discrepancies in the results of different studies.

## Figures and Tables

**Table 1.** Baseline characteristics of the study population

Characteristics	Total (n=1,067)	Survivors (n=212)	Death (n=578)	Lost to follow-up (n=277)
Age (yr)				
60-80	363 (34.0)	145 (68.4)	76 (13.1)	142 (51.3)
80-100	476 (44.6)	60 (28.3)	311 (53.8)	105 (37.9)
≥100	228 (21.4)	7 (3.3)	191 (33.0)	30 (10.8)
Sex				
Male	459 (43.0)	118 (55.7)	216 (37.4)	125 (45.1)
Female	608 (57.0)	94 (44.3)	362 (62.6)	152 (54.9)
Area of residence				
City/Town	245 (23.0)	40 (18.9)	102 (17.6)	103 (37.2)
Rural	822 (77.0)	172 (81.1)	476 (82.3)	174 (62.8)
Marital status				
Unmarried	9 (0.8)	0 (0.0)	7 (1.2)	2 (0.7)
Married	1,058 (99.2)	212 (100)	571 (98.8)	275 (99.3)
Economic status (RMB/yr)				
<10,000	509 (47.7)	102 (48.1)	301 (52.1)	106 (38.3)
≥10,000	558 (52.3)	110 (51.9)	277 (47.9)	171 (61.7)
Smoking status				
No	748 (70.1)	139 (65.6)	420 (72.7)	189 (68.2)
Yes	319 (29.9)	73 (34.4)	158 (27.3)	88 (31.8)
Drinking status				
No	797 (74.7)	146 (68.9)	446 (77.2)	205 (74.0)
Yes	270 (25.3)	66 (31.1)	132 (22.8)	72 (26.0)
SBP (mmHg)	142.54±22.04	141.00±21.60	143.87±22.76	140.95±20.68
DBP (mmHg)	78.68±11.52	78.77±11.37	78.47±12.04	79.07±10.49
BMI (kg/m^2^)	20.22±3.53	21.05±3.45	19.68±3.59	20.72±3.28
Blood urea nitrogen (mmol/L)	6.67±2.25	6.11±1.75	6.97±2.35	6.48±2.26
Plasma creatine (mmol/L)	87.10±33.86	80.71±23.57	88.74±37.42	88.58±32.20
Uric acid (μmol/L)	278.99±86.98	263.22±79.29	282.48±90.57	283.80±83.83
Plasma glucose (mmol/L)	5.43±1.89	5.24±1.83	5.44±1.81	5.53±2.10
Total cholesterol (mmol/L)	3.49±1.28	3.40±1.24	3.67±1.15	3.18±1.48
HDL cholesterol (mmol/L)	1.16±0.32	1.07±0.34	1.19±0.31	1.16±0.30
LDL cholesterol (mmol/L)	2.02±0.77	1.95±0.71	1.99±0.75	2.14±0.85
Triglyceride (mmol/L)	1.52±1.17	1.59±1.17	1.23±0.73	2.05±1.64

Values are presented as mean±standard deviation for continuous variables and number (%) for categorical variables. RMB, renminbi; SBP, systolic blood pressure; DBP, diastolic blood pressure; BMI, body mass index; HDL, high-density lipoprotein; LDL, low-density lipoprotein.

**Table 2.** Hazard ratios for all-cause mortality according to total cholesterol, HDL cholesterol, LDL cholesterol, and triglyceride quartiles in multivariate Cox regression analyses^1^

Quartiles		Individuals	Events, n (%)	Person-years	Model 1	p-value	Model 2	p-value	Model 3	p-value	Model 4	p-value
Total cholesterol (mmol/L)												
	Quartile 1 (<2.79)	268	113 (42.2)	1,480.17	1.00 (reference)		1.00 (reference)		1.00 (reference)		1.00 (reference)	
	Quartile 2 (2.79-3.58)	268	154 (57.5)	1,394.50	1.28 (0.99, 1.64)	0.056	1.27 (0.99, 1.63)	0.065	1.26 (0.98, 1.62)	0.077	1.23 (0.92, 1.65)	0.161
	Quartile 3 (3.58-4.29)	267	153 (57.3)	1,326.00	1.24 (0.96, 1.59)	0.097	1.23 (0.95, 1.58)	0.110	1.21 (0.94, 1.56)	0.134	1.44 (1.01, 2.06)	0.044
	Quartile 4 (≥4.29)	264	158 (59.8)	1,398.75	1.14 (0.89, 1.46)	0.312	1.13 (0.88, 1.45)	0.346	1.08 (0.84, 1.39)	0.558	1.51 (0.99, 2.32)	0.057
HDL cholesterol (mmol/L)												
	Quartile 1 (<0.94)	277	122 (44.0)	1,674.92	1.00 (reference)		1.00 (reference)		1.00 (reference)		1.00 (reference)	
	Quartile 2 (0.94-1.13)	262	153 (58.4)	1,258.42	1.32 (1.03, 1.68)	0.026	1.31 (1.03, 1.67)	0.030	1.28 (1.00, 1.63)	0.049	1.25 (0.96, 1.63)	0.097
	Quartile 3 (1.13-1.35)	246	133 (54.1)	1,260.75	1.13 (0.88, 1.45)	0.335	1.12 (0.88, 1.44)	0.354	1.13 (0.88, 1.46)	0.320	1.11 (0.84, 1.47)	0.475
	Quartile 4 (≥1.35)	282	170 (60.3)	1,405.33	1.14 (0.90, 1.45)	0.291	1.11 (0.87, 1.41)	0.389	1.11 (0.87, 1.42)	0.399	0.99 (0.74, 1.33)	0.950
LDL cholesterol (mmol/L)												
	Quartile 1 (<1.47)	260	151 (58.1)	1,312.75	1.00 (reference)		1.00 (reference)		1.00 (reference)		1.00 (reference)	
	Quartile 2 (1.47-1.91)	278	150 (54.0)	1,473.08	0.97 (0.77, 1.21)	0.762	0.97 (0.77, 1.22)	0.804	0.94 (0.74, 1.18)	0.570	0.84 (0.64, 1.09)	0.187
	Quartile 3 (1.91-2.47)	257	133 (51.7)	1,411.75	0.82 (0.65, 1.04)	0.099	0.82 (0.65, 1.05)	0.114	0.79 (0.62, 1.00)	0.051	0.68 (0.49, 0.94)	0.020
	Quartile 4 (≥2.47)	272	144 (52.9)	1,401.83	0.84 (0.66, 1.06)	0.137	0.84 (0.67, 1.07)	0.155	0.77 (0.61, 0.99)	0.037	0.71 (0.47, 1.07)	0.102
Triglyceride (mmol/L)												
	Quartile 1 (<0.84)	274	166 (60.6)	1,371.08	1.00 (reference)		1.00 (reference)		1.00 (reference)		1.00 (reference)	
	Quartile 2 (0.84-1.08)	256	152 (59.4)	1,346.92	0.81 (0.65, 1.02)	0.067	0.81 (0.65, 1.02)	0.069	0.80 (0.64, 1.01)	0.057	0.79 (0.62, 0.99)	0.045
	Quartile 3 (1.08-1.66)	274	166 (60.6)	1,353.58	0.92 (0.73, 1.14)	0.432	0.91 (0.73, 1.13)	0.377	0.84 (0.67, 1.06)	0.142	0.85 (0.66, 1.09)	0.192
	Quartile 4 (≥1.66)	263	94 (35.7)	1,527.83	0.59 (0.46, 0.77)	<0.001	0.59 (0.46, 0.77)	<0.001	0.51 (0.39, 0.68)	<0.001	0.55 (0.40, 0.75)	<0.001

Values are presented as hazard ratio (95% confidence interval). HDL, high-density lipoprotein; LDL, low-density lipoprotein; SBP, systolic blood pressure; DBP, diastolic blood pressure; BMI, body mass index.

1 Model 1: adjusted for age, sex, area of residence, marital status, economic status, smoking status, and drinking status; Model 2: further adjusted for SBP, DBP, and BMI based on model 1; Model 3: further adjusted for blood urea nitrogen, plasma creatine, uric acid, and plasma glucose based on model 2; Model 4: further adjusted for total cholesterol, HDL cholesterol, LDL cholesterol, and triglyceride levels based on model 3.

## SUPPLEMENTARY MATERIALS

Supplementary Material 1. Baseline characteristics of the study population according to quartiles of total cholesterol

Supplementary Material 2. Baseline characteristics of the study population according to quartiles of HDL cholesterol

Supplementary Material 3. Baseline characteristics of the study population according to quartiles of LDL cholesterol

Supplementary Material 4. Baseline characteristics of the study population according to quartiles of Triglyceride

Supplementary Material 5. Baseline characteristics of sex and age specific levels

Supplementary Material 6. Hazard ratios for all-cause mortality according to quartiles of total cholesterol, HDL cholesterol, LDL cholesterol and triglyceride in 60<age≤80 in multivariate Cox regression analyses

Supplementary Material 7. Hazard ratios for all-cause mortality according to quartiles of total cholesterol, HDL cholesterol, LDL cholesterol and triglyceride in 80<age≤100 in multivariate Cox regression analyses

Supplementary Material 8. Hazard ratios for all-cause mortality according to quartiles of total cholesterol, HDL cholesterol, LDL cholesterol and triglyceride in age>100 in multivariate Cox regression analyses
