# Supplementary material for: Blood lipid levels and all-cause mortality in older adults: the Chinese Longitudinal Healthy Longevity Survey 2008-2018
Source: Epidemiol Health. 2022 Jul 5;44:e2022054. doi: 10.4178/epih.e2022054 (PMC9754919; doi:10.4178/epih.e2022054)
Supplement: Supplementary Material 1. — Baseline characteristics of the study population according to quartiles of total cholesterol [file epih-44-e2022054-suppl1.docx]

**Supplementary Material 1.** Baseline characteristics of the study population according to quartiles of total cholesterol

|  | | | | | |
| --- | --- | --- | --- | --- | --- |
| haracteristics | Quartile 1(<2.79, n=268) | Quartile 2(2.79-3.58, n=268) | Quartile 3(3.58-4.29, n=267) | Quartile 4(≥4.29, n=264) | p Value |
| Age(years) |  |  |  |  | <0.001 |
| 60-80 | 118(44.03) | 94(35.07) | 80(29.96) | 71(26.89) |  |
| 80-100 | 115(42.91) | 121(45.15) | 119(44.57) | 121(45.83) |  |
| ≥100 | 35(13.06) | 53(19.78) | 68(25.47) | 72(27.27) |  |
| Sex |  |  |  |  | <0.001 |
| Male | 150(55.97) | 136(50.75) | 106(39.70) | 67(25.38) |  |
| Female | 118(44.03) | 132(49.25) | 161(60.30) | 197(74.62) |  |
| Category of residence |  |  |  |  | <0.001 |
| City/Town | 114(42.54) | 38(14.18) | 36(13.48) | 57(21.59) |  |
| Rural | 154(57.46) | 230(85.82) | 231(86.52) | 207(78.41) |  |
| Marital status |  |  |  |  | 0.753 |
| Unmarried | 3(1.12) | 3(1.12) | 2(0.75) | 1(0.38) |  |
| Married | 265(98.88) | 265(98.88) | 265(99.25) | 263(99.62) |  |
| Economic income (RMB) |  |  |  |  | <0.001 |
| <10000 | 93(34.70) | 139(51.87) | 139(52.06) | 138(52.27) |  |
| ≥10000 | 175(65.30) | 129(48.13) | 128(47.94) | 126(47.73) |  |
| Smoke |  |  |  |  | 0.015 |
| No | 173(64.55) | 182(67.91) | 190(71.16) | 203(76.89) |  |
| Yes | 95(35.45) | 86(32.09) | 77(28.84) | 61(23.11) |  |
| Drink |  |  |  |  | <0.001 |
| No | 203(75.75) | 177(66.04) | 199(74.53) | 218(82.58) |  |
| Yes | 65(24.25) | 91(33.96) | 68(25.47) | 46(17.42) |  |
| SBP(mmHg) | 143.08±20.53 | 141.04±22.31 | 142.36±23.26 | 143.70±22.01 | 0.541 |
| DBP(mmHg) | 77.40±11.62 | 78.25±11.81 | 80.11±11.15 | 78.98±11.36 | 0.046 |
| BMI(kg/m^2^) | 20.33±3.26 | 20.18±3.23 | 20.27±3.84 | 20.12±3.77 | 0.903 |
| Blood Urea Nitrogen(mmol/L) | 6.63±2.35 | 6.73±2.05 | 6.56±2.16 | 6.78±2.42 | 0.680 |
| Plasma creatine(mmol/L) | 93.54±40.70 | 84.56±29.13 | 83.39±30.59 | 86.91±33.10 | 0.002 |
| Urea acid(umol/L) | 285.35±95.90 | 280.18±82.37 | 268.84±84.23 | 281.61±84.33 | 0.147 |
| Plasma glucose(mmol/L) | 5.19±1.69 | 5.39±1.75 | 5.54±2.30 | 5.58±1.74 | 0.074 |
| HDL cholesterol(mmol/L) | 0.97±0.27 | 1.07±0.24 | 1.25±0.27 | 1.37±0.33 | <0.001 |
| LDL cholesterol(mmol/L) | 1.63±0.88 | 1.59±0.29 | 2.03±0.36 | 2.86±0.61 | <0.001 |
| Triglyceride(mmol/L) | 2.19±1.80 | 1.10±0.52 | 1.19±0.52 | 1.58±0.98 | <0.001 |
| SBP, systolic blood pressure; DBP, diastolic blood pressure; BMI, body mass index; HDL, high density lipoprotein; LDL, low density lipoprotein. Data are presented as mean ± SD (Standard Deviation) for continuous variables and n (%) for categorical variables. | | | | | |
