# Supplementary material for: Blood lipid levels and all-cause mortality in older adults: the Chinese Longitudinal Healthy Longevity Survey 2008-2018
Source: Epidemiol Health. 2022 Jul 5;44:e2022054. doi: 10.4178/epih.e2022054 (PMC9754919; doi:10.4178/epih.e2022054)
Supplement: Supplementary Material 2. — Baseline characteristics of the study population according to quartiles of HDL cholesterol [file epih-44-e2022054-suppl2.docx]

**Supplementary** **Material 2.** Baseline characteristics of the study population according to quartiles of HDL cholesterol

|  | | | | | |
| --- | --- | --- | --- | --- | --- |
| Characteristics | Quartile 1(<0.94, n=277) | Quartile 2(0.94-1.13, n=262) | Quartile 3(1.13-1.35, n=246) | Quartile 4(≥1.35, n=282) | p Value |
| Age(years) |  |  |  |  | <0.001 |
| 60-80 | 133(48.01) | 89(33.97) | 79(32.11) | 62(21.99) |  |
| 80-100 | 113(40.79) | 116(44.27) | 113(45.93) | 134(47.52) |  |
| ≥100 | 31(11.19) | 57(21.76) | 54(21.95) | 86(30.50) |  |
| Sex |  |  |  |  | <0.001 |
| Male | 149(53.79) | 122(46.56) | 100(40.65) | 88(31.21) |  |
| Female | 128(46.21) | 140(53.44) | 146(59.35) | 194(68.79) |  |
| Category of residence |  |  |  |  | 0.872 |
| City/Town | 68(24.55) | 59(22.52) | 53(21.54) | 65(23.05) |  |
| Rural | 209(75.45) | 203(77.48) | 193(78.46) | 217(76.95) |  |
| Marital status |  |  |  |  | 0.327 |
| Unmarried | 1(0.36) | 4(1.53) | 3(1.22) | 1(0.35) |  |
| Married | 276(99.64) | 258(98.47) | 243(98.78) | 281(99.65) |  |
| Economic income (RMB) |  |  |  |  | 0.009 |
| <10000 | 112(40.43) | 144(54.96) | 116(47.15) | 137(48.58) |  |
| ≥10000 | 165(59.57) | 118(45.04) | 130(52.85) | 145(51.42) |  |
| Smoke |  |  |  |  | 0.008 |
| No | 176(63.54) | 180(68.70) | 176(71.54) | 216(76.60) |  |
| Yes | 101(36.46) | 82(31.30) | 70(28.46) | 66(23.40) |  |
| Drink |  |  |  |  | 0.222 |
| No | 199(71.84) | 191(72.90) | 195(79.27) | 212(75.18) |  |
| Yes | 78(28.16) | 71(27.10) | 51(20.73) | 70(24.82) |  |
| SBP(mmHg) | 143.16±22.04 | 142.60±21.97 | 143.18±23.45 | 141.32±20.87 | 0.730 |
| DBP(mmHg) | 77.71±11.94 | 79.19±11.17 | 80.05±11.81 | 77.98±11.06 | 0.072 |
| BMI(kg/m2) | 20.70±3.04 | 20.22±3.75 | 20.57±3.68 | 19.47±3.53 | <0.001 |
| Blood Urea Nitrogen(mmol/L) | 6.37±2.29 | 6.83±2.36 | 6.43±2.02 | 7.03±2.24 | 0.001 |
| Plasma creatine(mmol/L) | 88.49±36.60 | 91.02±35.94 | 86.31±31.33 | 82.80±30.70 | 0.034 |
| Urea acid(umol/L) | 277.91±87.53 | 288.36±94.06 | 273.32±79.25 | 276.30±85.82 | 0.222 |
| Plasma glucose(mmol/L) | 5.63±2.64 | 5.46±1.79 | 5.29±1.47 | 5.31±1.33 | 0.132 |
| Total cholesterol(mmol/L) | 2.88±1.16 | 3.23±1.06 | 3.66±1.15 | 4.19±1.31 | <0.001 |
| LDL cholesterol(mmol/L) | 1.71±0.70 | 1.96±0.68 | 2.18±0.70 | 2.24±0.87 | <0.001 |
| Triglyceride(mmol/L) | 1.57±1.20 | 1.57±1.10 | 1.55±1.16 | 1.39±1.21 | 0.206 |
| SBP, systolic blood pressure; DBP, diastolic blood pressure; BMI, body mass index; HDL, high density lipoprotein; LDL, low density lipoprotein. Data are presented as mean ± SD (Standard Deviation) for continuous variables and n (%) for categorical variables. | | | | | |
