# Supplementary material for: Blood lipid levels and all-cause mortality in older adults: the Chinese Longitudinal Healthy Longevity Survey 2008-2018
Source: Epidemiol Health. 2022 Jul 5;44:e2022054. doi: 10.4178/epih.e2022054 (PMC9754919; doi:10.4178/epih.e2022054)
Supplement: Supplementary Material 3. — Baseline characteristics of the study population according to quartiles of LDL cholesterol [file epih-44-e2022054-suppl3.docx]

**Supplementary** **Material 3**. Baseline characteristics of the study population according to quartiles of LDL cholesterol

|  | | | | | |
| --- | --- | --- | --- | --- | --- |
| Characteristics | Quartile 1(<1.47, n=260) | Quartile 2(1.47-1.91, n=278) | Quartile 3(1.91-2.47, n=257) | Quartile 4(≥2.47, n=272) | p Value |
| Age(years) |  |  |  |  | 0.749 |
| 60-80 | 86(33.08) | 98(35.25) | 92(35.80) | 87(31.99) |  |
| 80-100 | 122(46.92) | 126(45.32) | 110(42.80) | 118(43.38) |  |
| ≥100 | 52(20.00) | 54(19.42) | 55(21.40) | 67(24.63) |  |
| Sex |  |  |  |  | <0.001 |
| Male | 138(53.08) | 144(51.80) | 90(35.02) | 87(31.99) |  |
| Female | 122(46.92) | 134(48.20) | 167(64.98) | 185(68.01) |  |
| Category of residence |  |  |  |  | <0.001 |
| City/Town | 42(16.15) | 47(16.91) | 59(22.96) | 97(35.66) |  |
| Rural | 218(83.85) | 231(83.09) | 198(77.04) | 175(64.34) |  |
| Marital status |  |  |  |  | 0.703 |
| Unmarried | 3(1.15) | 2(0.72) | 3(1.17) | 1(0.37) |  |
| Married | 257(98.85) | 276(99.28) | 254(98.83) | 271(99.63) |  |
| Economic income (RMB) |  |  |  |  | 0.127 |
| <10000 | 113(43.46) | 147(52.88) | 126(49.03) | 123(45.22) |  |
| ≥10000 | 147(56.54) | 131(47.12) | 131(50.97) | 149(54.78) |  |
| Smoke |  |  |  |  | 0.301 |
| No | 174(66.92) | 189(67.99) | 187(72.76) | 198(72.79) |  |
| Yes | 86(33.08) | 89(32.01) | 70(27.24) | 74(27.21) |  |
| Drink |  |  |  |  | <0.001 |
| No | 179(68.85) | 192(69.06) | 205(79.77) | 221(81.25) |  |
| Yes | 81(31.15) | 86(30.94) | 52(20.23) | 51(18.75) |  |
| SBP(mmHg) | 142.23±21.71 | 141.33±22.60 | 142.14±23.07 | 144.45±20.74 | 0.390 |
| DBP(mmHg) | 78.22±12.25 | 78.30±12.28 | 79.61±10.47 | 78.64±10.94 | 0.494 |
| BMI(kg/m2) | 19.76±2.95 | 20.23±3.15 | 20.30±3.60 | 20.59±4.24 | 0.056 |
| Blood Urea Nitrogen(mmol/L) | 6.91±2.36 | 6.73±2.03 | 6.49±1.93 | 6.56±2.60 | 0.138 |
| Plasma creatine(mmol/L) | 85.93±39.60 | 86.28±29.26 | 84.36±29.02 | 91.66±36.21 | 0.069 |
| Urea acid(umol/L) | 270.15±89.81 | 273.21±77.33 | 273.67±82.69 | 298.40±94.68 | <0.001 |
| Plasma glucose(mmol/L) | 5.17±1.86 | 5.36±1.42 | 5.55±2.42 | 5.61±1.75 | 0.031 |
| Total cholesterol(mmol/L) | 2.57±0.77 | 3.26±0.83 | 3.73±1.01 | 4.38±1.57 | <0.001 |
| HDL cholesterol(mmol/L) | 1.03±0.32 | 1.17±0.32 | 1.17±0.30 | 1.25±0.29 | <0.001 |
| Triglyceride(mmol/L) | 0.96±0.50 | 1.25±0.79 | 1.57±1.10 | 2.28±1.56 | <0.001 |
| SBP, systolic blood pressure; DBP, diastolic blood pressure; BMI, body mass index; HDL, high density lipoprotein; LDL, low density lipoprotein. Data are presented as mean ± SD (Standard Deviation) for continuous variables and n (%) for categorical variables. | | | | | |
