# Supplementary material for: Blood lipid levels and all-cause mortality in older adults: the Chinese Longitudinal Healthy Longevity Survey 2008-2018
Source: Epidemiol Health. 2022 Jul 5;44:e2022054. doi: 10.4178/epih.e2022054 (PMC9754919; doi:10.4178/epih.e2022054)
Supplement: Supplementary Material 4. — Baseline characteristics of the study population according to quartiles of Triglyceride [file epih-44-e2022054-suppl4.docx]

**Supplementary** **Material 4.** Baseline characteristics of the study population according to quartiles of Triglyceride

|  | | | | | |
| --- | --- | --- | --- | --- | --- |
| Characteristics | Quartile 1(<0.84, n=274) | Quartile 2(0.84-1.08, n=256) | Quartile 3(1.08-1.66, n=274) | Quartile 4(≥1.66, n=263) | p Value |
| Age(years) |  |  |  |  | <0.001 |
| 60-80 | 86(31.39) | 72(28.13) | 78(28.47) | 127(48.29) |  |
| 80-100 | 131(47.81) | 118(46.09) | 130(47.45) | 97(36.88) |  |
| ≥100 | 57(20.80) | 66(25.78) | 66(24.09) | 39(14.83) |  |
| Sex |  |  |  |  | <0.001 |
| Male | 147(53.65) | 98(38.28) | 98(35.77) | 116(44.11) |  |
| Female | 127(46.35) | 158(61.72) | 176(64.23) | 147(55.89) |  |
| Category of residence |  |  |  |  | <0.001 |
| City/Town | 41(14.96) | 48(18.75) | 35(12.77) | 121(46.01) |  |
| Rural | 233(85.04) | 208(81.25) | 239(87.23) | 142(53.99) |  |
| Marital status |  |  |  |  | 0.494 |
| Unmarried | 2(0.73) | 4(1.56) | 1(0.36) | 2(0.76) |  |
| Married | 272(99.27) | 252(98.44) | 273(99.64) | 261(99.24) |  |
| Economic income (RMB) |  |  |  |  | 0.001 |
| <10000 | 122(44.53) | 141(55.08) | 144(52.55) | 102(38.78) |  |
| ≥10000 | 152(55.47) | 115(44.92) | 130(47.45) | 161(61.22) |  |
| Smoke |  |  |  |  | 0.001 |
| No | 169(61.68) | 194(75.78) | 205(74.82) | 180(68.44) |  |
| Yes | 105(38.32) | 62(24.22) | 69(25.18) | 83(31.56) |  |
| Drink |  |  |  |  | 0.001 |
| No | 180(65.69) | 194(75.78) | 218(79.56) | 205(77.95) |  |
| Yes | 94(34.31) | 62(24.22) | 56(20.44) | 58(22.05) |  |
| SBP(mmHg) | 140.58±21.40 | 143.02±23.31 | 142.86±22.53 | 143.79±20.86 | 0.363 |
| DBP(mmHg) | 78.26±10.85 | 78.58±12.43 | 79.10±11.06 | 78.79±11.78 | 0.853 |
| BMI(kg/m2) | 19.97±2.71 | 20.14±3.62 | 19.86±3.31 | 20.95±4.27 | 0.001 |
| Blood Urea Nitrogen(mmol/L) | 7.11±2.28 | 6.87±2.30 | 6.55±2.11 | 6.16±2.20 | <0.001 |
| Plasma creatine(mmol/L) | 85.27±35.58 | 82.10±32.82 | 85.53±32.55 | 95.52±33.05 | <0.001 |
| Urea acid(umol/L) | 268.85±85.72 | 267.19±78.50 | 274.86±87.28 | 305.35±90.74 | <0.001 |
| Plasma glucose(mmol/L) | 5.11±1.63 | 5.12±1.23 | 5.50±1.89 | 5.98±2.47 | <0.001 |
| Total cholesterol(mmol/L) | 3.16±0.83 | 3.73±0.90 | 3.99±0.97 | 3.08±1.88 | <0.001 |
| HDL cholesterol(mmol/L) | 1.13±0.33 | 1.24±0.32 | 1.19±0.30 | 1.08±0.30 | <0.001 |
| LDL cholesterol(mmol/L) | 1.50±0.51 | 1.89±0.59 | 2.18±0.70 | 2.54±0.84 | <0.001 |
| SBP, systolic blood pressure; DBP, diastolic blood pressure; BMI, body mass index; HDL, high density lipoprotein; LDL, low density lipoprotein. Data are presented as mean ± SD (Standard Deviation) for continuous variables and n (%) for categorical variables. | | | | | |
