# Supplementary material for: Blood lipid levels and all-cause mortality in older adults: the Chinese Longitudinal Healthy Longevity Survey 2008-2018
Source: Epidemiol Health. 2022 Jul 5;44:e2022054. doi: 10.4178/epih.e2022054 (PMC9754919; doi:10.4178/epih.e2022054)
Supplement: Supplementary Material 5. — Baseline characteristics of sex and age specific levels [file epih-44-e2022054-suppl5.docx]

**Supplementary Material 5.** Baseline characteristics of sex and age specific levels

|  | | | | | | | | | | | | |
| --- | --- | --- | --- | --- | --- | --- | --- | --- | --- | --- | --- | --- |
| Characteristics | Male | | | | |  | Female | | | | |  |
|  | 60-69 | 70-79 | 80-89 | 90-99 | ≥100 |  | 60-69 | 70-79 | 80-89 | 90-99 | ≥100 |  |
| Category of residence |  |  |  |  |  |  |  |  |  |  |  |  |
| City/Town | 37(30.33) | 32(30.77) | 27(21.60) | 16(21.92) | 7(20.00) |  | 13(18.57) | 13(19.40) | 40(31.01) | 36(24.16) | 24(12.44) |  |
| Rural | 85(69.67) | 72(69.23) | 98(78.40) | 57(78.08) | 28(80.00) |  | 57(81.43) | 54(80.60) | 89(68.99) | 113(75.84) | 169(87.56) |  |
| Marital status |  |  |  |  |  |  |  |  |  |  |  |  |
| Unmarried | 1(0.82) | 2(1.92) | 3(2.40) | 2(2.74) | 1(2.86) |  | 0 | 0 | 0 | 0 | 0 |  |
| Married | 121(99.18) | 102(98.08) | 122(97.60) | 71(97.26) | 34(97.14) |  | 70(100.00) | 67(100.00) | 129(100.00) | 149(100.00) | 193(100.00) |  |
| Economic income (RMB) |  |  |  |  |  |  |  |  |  |  |  |  |
| <10000 | 46(37.70) | 53(50.96) | 69(55.20) | 43(58.90) | 16(45.71) |  | 29(41.43) | 34(50.75) | 59(45.74) | 70(46.98) | 90(46.63) |  |
| ≥10000 | 76(62.30) | 51(49.04) | 56(44.80) | 30(41.10) | 19(54.29) |  | 41(58.57) | 33(49.25) | 70(54.26) | 79(53.02) | 103(53.37) |  |
| Smoke |  |  |  |  |  |  |  |  |  |  |  |  |
| No | 47(38.52) | 36(34.62) | 52(41.60) | 37(50.68) | 20(57.14) |  | 64(91.43) | 66(98.51) | 117(90.70) | 136(91.28) | 173(89.64) |  |
| Yes | 75(61.48) | 68(65.38) | 73(58.40) | 36(49.32) | 15(42.86) |  | 6(8.57) | 1(1.49) | 12(9.30) | 13(8.72) | 20(10.36) |  |
| Drink |  |  |  |  |  |  |  |  |  |  |  |  |
| No | 62(50.82) | 61(58.65) | 75(60.00) | 44(60.27) | 19(54.29) |  | 61(87.14) | 64(95.52) | 114(88.37) | 133(89.26) | 164(84.97) |  |
| Yes | 60(49.18) | 43(41.35) | 50(40.00) | 29(39.73) | 16(45.71) |  | 9(12.86) | 3(4.48) | 15(11.63) | 16(10.74) | 29(15.03) |  |
| SBP(mmHg) | 136.65±18.15 | 141.09±21.19 | 144.18±22.00 | 144.55±22.08 | 145.90±21.72 |  | 141.41±20.26 | 146.20±26.81 | 143.95±21.48 | 145.03±25.28 | 140.90±20.79 |  |
| DBP(mmHg) | 80.43±11.77 | 78.82±11.22 | 77.39±11.73 | 78.36±12.56 | 80.46±8.41 |  | 79.98±10.80 | 82.56±13.79 | 78.01±10.96 | 79.31±11.24 | 76.30±10.97 |  |
| BMI(kg/m^2^) | 22.31±3.73 | 21.53±2.89 | 20.49±2.75 | 19.91±2.60 | 19.36±3.79 |  | 21.63±3.26 | 20.95±3.11 | 19.84±3.10 | 18.88±3.41 | 18.84±3.91 |  |
| Blood Urea Nitrogen(mmol/L) | 6.00±1.67 | 6.21±1.58 | 6.98±2.15 | 7.28±2.28 | 7.12±1.91 |  | 5.46±1.31 | 5.85±2.16 | 6.56±1.84 | 6.95±2.51 | 7.43±2.84 |  |
| Plasma creatine(mmol/L) | 84.98±21.41 | 93.30±25.81 | 105.62±41.42 | 101.58±37.03 | 90.74±31.93 |  | 63.60±13.31 | 80.18±30.31 | 80.02±31.70 | 86.38±36.80 | 83.19±35.03 |  |
| Urea acid(umol/L) | 291.71±86.23 | 296.85±79.99 | 309.02±94.63 | 329.63±94.39 | 322.54±77.41 |  | 220.49±61.50 | 244.12±80.54 | 246.00±71.26 | 277.92±85.07 | 271.05±80.60 |  |
| Plasma glucose(mmol/L) | 5.39±1.56 | 5.64±2.15 | 5.28±2.19 | 5.82±2.28 | 5.56±1.44 |  | 5.47±2.83 | 5.77±2.19 | 5.12±1.14 | 5.46±1.90 | 5.29±1.42 |  |
| Total cholesterol(mmol/L) | 2.93±1.18 | 3.03±1.38 | 3.06±1.29 | 3.57±1.00 | 3.54±0.85 |  | 3.40±1.16 | 3.88±1.34 | 3.17±1.39 | 4.09±1.03 | 3.98±1.13 |  |
| HDL cholesterol(mmol/L) | 1.05±0.32 | 1.09±0.30 | 1.11±0.33 | 1.18±0.31 | 1.13±0.23 |  | 1.04±0.29 | 1.13±0.35 | 1.14±0.31 | 1.30±0.30 | 1.26±0.30 |  |
| LDL cholesterol(mmol/L) | 1.89±0.84 | 1.93±0.73 | 1.78±0.66 | 1.92±0.76 | 1.88±0.67 |  | 2.01±0.60 | 2.32±0.79 | 2.05±0.76 | 2.18±0.78 | 2.14±0.83 |  |
| Triglyceride(mmol/L) | 1.89±1.63 | 1.75±1.31 | 1.38±1.09 | 1.14±0.55 | 1.15±0.58 |  | 1.91±1.51 | 1.90±1.50 | 1.83±1.45 | 1.20±0.53 | 1.22±0.57 |  |
| SBP, systolic blood pressure; DBP, diastolic blood pressure; BMI, body mass index; HDL, high density lipoprotein; LDL, low density lipoprotein. Data are presented as mean ± SD (Standard Deviation) for continuous variables and n (%) for categorical variables. | | | | | | | | | | | |  |
