# Supplementary material for: Blood lipid levels and all-cause mortality in older adults: the Chinese Longitudinal Healthy Longevity Survey 2008-2018
Source: Epidemiol Health. 2022 Jul 5;44:e2022054. doi: 10.4178/epih.e2022054 (PMC9754919; doi:10.4178/epih.e2022054)
Supplement: Supplementary Material 6. — Hazard ratios for all-cause mortality according to quartiles of total cholesterol, HDL cholesterol, LDL cholesterol and triglyceride in 60<age≤80 in multivariate Cox regression analyses [file epih-44-e2022054-suppl6.docx]

**Supplementary Material 6.** Hazard ratios for all-cause mortality according to quartiles of total cholesterol, HDL cholesterol, LDL cholesterol and triglyceride in 60<age≤80 in multivariate Cox regression analyses

|  | | | | | | | | | | | | | | |
| --- | --- | --- | --- | --- | --- | --- | --- | --- | --- | --- | --- | --- | --- | --- |
| Quartiles | Individuals | Events (%) | Person-years | Model 1 | |  | Model 2 | |  | Model 3 | |  | Model 4 | |
|  |  |  |  | HR (95% CI) | p value |  | HR (95% CI) | p value |  | HR (95% CI) | p value |  | HR (95% CI) | p value |
| Total cholesterol(mmol/L) |  |  |  |  |  |  |  |  |  |  |  |  |  |  |
| Quartile 1(<2.79) | 118 | 20(16.95) | 727.75 | Reference |  |  | Reference |  |  | Reference |  |  | Reference |  |
| Quartile 2(2.79-3.58) | 94 | 23(24.47) | 665.08 | 1.19(0.63-2.27) | 0.590 |  | 1.21(0.64-2.30) | 0.562 |  | 1.12(0.58-2.20) | 0.731 |  | 0.98(0.45-2.13) | 0.957 |
| Quartile 3(3.58-4.29) | 80 | 19(23.75) | 590.00 | 1.04(0.52-2.06) | 0.917 |  | 0.98(0.49-1.96) | 0.957 |  | 0.95(0.47-1.93) | 0.888 |  | 1.14(0.43-3.05) | 0.787 |
| Quartile 4(≥4.29) | 71 | 14(19.72) | 551.42 | 0.83(0.40-1.74) | 0.621 |  | 0.72(0.34-1.54) | 0.401 |  | 0.57(0.26-1.27) | 0.170 |  | 0.80(0.25-2.56) | 0.712 |
| HDL cholesterol(mmol/L) |  |  |  |  |  |  |  |  |  |  |  |  |  |  |
| Quartile 1(<0.94) | 133 | 22(16.54) | 975.00 | Reference |  |  | Reference |  |  | Reference |  |  | Reference |  |
| Quartile 2(0.94-1.13) | 89 | 27(30.34) | 551.42 | 2.12(1.19-3.75) | 0.010 |  | 2.27(1.27-4.06) | 0.006 |  | 2.23(1.22-4.07) | 0.009 |  | 2.24(1.20-4.20) | 0.011 |
| Quartile 3(1.13-1.35) | 79 | 17(21.52) | 537.08 | 1.34(0.71-2.53) | 0.375 |  | 1.30(0.68-2.49) | 0.426 |  | 1.23(0.63-2.42) | 0.548 |  | 1.30(0.63-2.67) | 0.472 |
| Quartile 4(≥1.35) | 62 | 10(16.13) | 470.75 | 0.96(0.45-2.05) | 0.923 |  | 0.89(0.42-1.92) | 0.773 |  | 0.93(0.43-2.04) | 0.864 |  | 1.00(0.42-2.40) | 0.998 |
| LDL cholesterol(mmol/L) |  |  |  |  |  |  |  |  |  |  |  |  |  |  |
| Quartile 1(<1.47) | 86 | 21(24.42) | 573.08 | Reference |  |  | Reference |  |  | Reference |  |  | Reference |  |
| Quartile 2(1.47-1.91) | 98 | 23(23.47) | 693.67 | 1.00(0.55-1.83) | 1.000 |  | 0.93(0.51-1.71) | 0.826 |  | 0.83(0.45-1.54) | 0.552 |  | 0.89(0.41-1.92) | 0.764 |
| Quartile 3(1.91-2.47) | 92 | 18(19.57) | 682.58 | 0.66(0.35-1.26) | 0.209 |  | 0.60(0.31-1.15) | 0.125 |  | 0.51(0.26-1.00) | 0.051 |  | 0.54(0.20-1.45) | 0.223 |
| Quartile 4(≥2.47) | 87 | 14(16.09) | 584.92 | 0.73(0.36-1.48) | 0.384 |  | 0.69(0.34-1.41) | 0.305 |  | 0.49(0.23-1.05) | 0.066 |  | 0.62(0.19-1.99) | 0.420 |
| Triglyceride(mmol/L) |  |  |  |  |  |  |  |  |  |  |  |  |  |  |
| Quartile 1(<0.84) | 86 | 22(25.58) | 611.75 | Reference |  |  | Reference |  |  | Reference |  |  | Reference |  |
| Quartile 2(0.84-1.08) | 72 | 18(25.00) | 527.50 | 1.01(0.53-1.91) | 0.981 |  | 1.00(0.53-1.91) | 0.989 |  | 1.01(0.53-1.93) | 0.968 |  | 1.13(0.57-2.24) | 0.723 |
| Quartile 3(1.08-1.66) | 78 | 17(21.79) | 554.25 | 0.89(0.47-1.69) | 0.731 |  | 0.86(0.46-1.64) | 0.656 |  | 0.74(0.39-1.43) | 0.375 |  | 0.98(0.46-2.08) | 0.953 |
| Quartile 4(≥1.66) | 127 | 19(14.96) | 840.75 | 0.70(0.36-1.35) | 0.288 |  | 0.73(0.37-1.42) | 0.350 |  | 0.51(0.25-1.07) | 0.075 |  | 0.71(0.30-1.68) | 0.432 |
| HDL, high density lipoprotein; LDL, low density lipoprotein; SBP, systolic blood pressure; DBP, diastolic blood pressure; BMI, body mass index. Model 1: adjusted for age, sex, category of residence, marital status, economic income, smoke and drink; Model 2: further adjusted for SBP, DBP and BMI based on model 1; Model 3: further adjusted for blood urea nitrogen, plasma creatine, urea acid and plasma glucose based on model 2; Model 4: further adjusted for total cholesterol, HDL cholesterol, LDL cholesterol and triglyceride based on model 3. | | | | | | | | | | | | | | |
