# Supplementary material for: Blood lipid levels and all-cause mortality in older adults: the Chinese Longitudinal Healthy Longevity Survey 2008-2018
Source: Epidemiol Health. 2022 Jul 5;44:e2022054. doi: 10.4178/epih.e2022054 (PMC9754919; doi:10.4178/epih.e2022054)
Supplement: Supplementary Material 7. — Hazard ratios for all-cause mortality according to quartiles of total cholesterol, HDL cholesterol, LDL cholesterol and triglyceride in 80<age≤100 in multivariate Cox regression analyses [file epih-44-e2022054-suppl7.docx]

**Supplementary Material 7.** Hazard ratios for all-cause mortality according to quartiles of total cholesterol, HDL cholesterol, LDL cholesterol and triglyceride in 80<age≤100 in multivariate Cox regression analyses

| Quartiles | Individuals | Events (%) | Person-years | Model 1 | |  | Model 2 | |  | Model 3 | |  | Model 4 | |
| --- | --- | --- | --- | --- | --- | --- | --- | --- | --- | --- | --- | --- | --- | --- |
|  |  |  |  | HR (95% CI) | p value |  | HR (95% CI) | p value |  | HR (95% CI) | p value |  | HR (95% CI) | p value |
| Total cholesterol(mmol/L) |  |  |  |  |  |  |  |  |  |  |  |  |  |  |
| Quartile 1(<2.79) | 115 | 64(55.65) | 638.00 | Reference |  |  | Reference |  |  | Reference |  |  | Reference |  |
| Quartile 2(2.79-3.58) | 121 | 86(71.07) | 562.67 | 1.38(0.98-1.95) | 0.062 |  | 1.39(0.99-1.95) | 0.059 |  | 1.40(1.00-1.97) | 0.052 |  | 1.62(1.08-2.43) | 0.019 |
| Quartile 3(3.58-4.29) | 119 | 77(64.71) | 520.33 | 1.43(1.02-2.01) | 0.040 |  | 1.42(1.01-1.99) | 0.044 |  | 1.47(1.04-2.06) | 0.028 |  | 1.83(1.14-2.91) | 0.012 |
| Quartile 4(≥4.29) | 121 | 84(69.42) | 599.25 | 1.35(0.96-1.89) | 0.084 |  | 1.34(0.96-1.88) | 0.085 |  | 1.30(0.92-1.82) | 0.135 |  | 1.56(0.90-2.71) | 0.113 |
| HDL cholesterol(mmol/L) |  |  |  |  |  |  |  |  |  |  |  |  |  |  |
| Quartile 1(<0.94) | 113 | 74(65.49) | 583.17 | Reference |  |  | Reference |  |  | Reference |  |  | Reference |  |
| Quartile 2(0.94-1.13) | 116 | 77(66.38) | 538.08 | 1.11(0.80-1.54) | 0.533 |  | 1.07(0.77-1.49) | 0.684 |  | 1.04(0.75-1.45) | 0.811 |  | 0.95(0.66-1.37) | 0.792 |
| Quartile 3(1.13-1.35) | 113 | 69(61.06) | 557.75 | 0.97(0.70-1.35) | 0.856 |  | 0.94(0.67-1.30) | 0.696 |  | 0.96(0.69-1.33) | 0.791 |  | 0.79(0.53-1.15) | 0.220 |
| Quartile 4(≥1.35) | 134 | 91(67.91) | 641.25 | 1.18(0.86-1.62) | 0.297 |  | 1.12(0.82-1.55) | 0.475 |  | 1.15(0.84-1.59) | 0.386 |  | 0.98(0.67-1.45) | 0.936 |
| LDL cholesterol(mmol/L) |  |  |  |  |  |  |  |  |  |  |  |  |  |  |
| Quartile 1(<1.47) | 122 | 86(70.49) | 567.25 | Reference |  |  | Reference |  |  | Reference |  |  | Reference |  |
| Quartile 2(1.47-1.91) | 126 | 80(63.49) | 649.25 | 0.80(0.59-1.08) | 0.148 |  | 0.80(0.58-1.08) | 0.144 |  | 0.79(0.58-1.08) | 0.144 |  | 0.67(0.47-0.95) | 0.025 |
| Quartile 3(1.91-2.47) | 110 | 70(63.64) | 525.08 | 0.88(0.64-1.22) | 0.439 |  | 0.90(0.65-1.25) | 0.523 |  | 0.88(0.64-1.23) | 0.470 |  | 0.81(0.53-1.22) | 0.311 |
| Quartile 4(≥2.47) | 118 | 75(63.56) | 578.67 | 0.89(0.65-1.22) | 0.467 |  | 0.90(0.65-1.24) | 0.517 |  | 0.85(0.61-1.18) | 0.334 |  | 0.89(0.52-1.52) | 0.673 |
| Triglyceride(mmol/L) |  |  |  |  |  |  |  |  |  |  |  |  |  |  |
| Quartile 1(<0.84) | 131 | 97(74.05) | 589.83 | Reference |  |  | Reference |  |  | Reference |  |  | Reference |  |
| Quartile 2(0.84-1.08) | 118 | 80(67.80) | 594.08 | 0.77(0.57-1.05) | 0.094 |  | 0.77(0.57-1.05) | 0.096 |  | 0.76(0.56-1.04) | 0.090 |  | 0.73(0.52-1.01) | 0.054 |
| Quartile 3(1.08-1.66) | 130 | 89(68.46) | 593.67 | 0.89(0.66-1.19) | 0.432 |  | 0.88(0.65-1.18) | 0.392 |  | 0.82(0.60-1.12) | 0.208 |  | 0.76(0.54-1.06) | 0.105 |
| Quartile 4(≥1.66) | 97 | 45(46.39) | 542.67 | 0.51(0.35-0.75) | <0.001 |  | 0.53(0.36-0.76) | 0.001 |  | 0.46(0.31-0.69) | <0.001 |  | 0.49(0.32-0.77) | 0.002 |
| HDL, high density lipoprotein; LDL, low density lipoprotein; SBP, systolic blood pressure; DBP, diastolic blood pressure; BMI, body mass index. Model 1: adjusted for age, sex, category of residence, marital status, economic income, smoke and drink; Model 2: further adjusted for SBP, DBP and BMI based on model 1; Model 3: further adjusted for blood urea nitrogen, plasma creatine, urea acid and plasma glucose based on model 2; Model 4: further adjusted for total cholesterol, HDL cholesterol, LDL cholesterol and triglyceride based on model 3. | | | | | | | | | | | | | | |
