# Supplementary material for: Blood lipid levels and all-cause mortality in older adults: the Chinese Longitudinal Healthy Longevity Survey 2008-2018
Source: Epidemiol Health. 2022 Jul 5;44:e2022054. doi: 10.4178/epih.e2022054 (PMC9754919; doi:10.4178/epih.e2022054)
Supplement: Supplementary Material 8. — Hazard ratios for all-cause mortality according to quartiles of total cholesterol, HDL cholesterol, LDL cholesterol and triglyceride in age>100 in multivariate Cox regression analyses [file epih-44-e2022054-suppl8.docx]

**Supplementary Material 8.** Hazard ratios for all-cause mortality according to quartiles of total cholesterol, HDL cholesterol, LDL cholesterol and triglyceride in age>100 in multivariate Cox regression analyses

Table

| Quartiles | Individuals | Events (%) | Person-years | Model 1 | |  | Model 2 | |  | Model 3 | |  | Model 4 | |
| --- | --- | --- | --- | --- | --- | --- | --- | --- | --- | --- | --- | --- | --- | --- |
|  |  |  |  | HR (95% CI) | p value |  | HR (95% CI) | p value |  | HR (95% CI) | p value |  | HR (95% CI) | p value |
| Total cholesterol(mmol/L) |  |  |  |  |  |  |  |  |  |  |  |  |  |  |
| Quartile 1(<2.79) | 35 | 29(82.86) | 114.42 | Reference |  |  | Reference |  |  | Reference |  |  | Reference |  |
| Quartile 2(2.79-3.58) | 53 | 45(84.91) | 166.75 | 1.00(0.62-1.63) | 0.984 |  | 0.95(0.58-1.56) | 0.851 |  | 0.86(0.52-1.43) | 0.565 |  | 0.98(0.47-2.02) | 0.954 |
| Quartile 3(3.58-4.29) | 68 | 57(83.82) | 215.67 | 1.03(0.66-1.63) | 0.887 |  | 1.04(0.65-1.66) | 0.886 |  | 0.90(0.54-1.49) | 0.680 |  | 1.63(0.62-4.31) | 0.321 |
| Quartile 4(≥4.29) | 72 | 60(83.33) | 248.08 | 0.94(0.60-1.49) | 0.807 |  | 0.92(0.58-1.47) | 0.725 |  | 0.76(0.46-1.28) | 0.301 |  | 2.25(0.70-7.23) | 0.174 |
| HDL cholesterol(mmol/L) |  |  |  |  |  |  |  |  |  |  |  |  |  |  |
| Quartile 1(<0.94) | 31 | 26(83.87) | 116.75 | Reference |  |  | Reference |  |  | Reference |  |  | Reference |  |
| Quartile 2(0.94-1.13) | 57 | 49(85.96) | 168.92 | 1.40(0.86-2.28) | 0.171 |  | 1.42(0.87-2.30) | 0.159 |  | 1.32(0.80-2.16) | 0.277 |  | 1.35(0.73-2.50) | 0.342 |
| Quartile 3(1.13-1.35) | 54 | 47(87.04) | 165.92 | 1.31(0.80-2.12) | 0.280 |  | 1.32(0.81-2.15) | 0.272 |  | 1.28(0.78-2.10) | 0.327 |  | 1.40(0.71-2.72) | 0.329 |
| Quartile 4(≥1.35) | 86 | 69(80.23) | 293.33 | 1.07(0.68-1.69) | 0.778 |  | 1.04(0.65-1.64) | 0.881 |  | 0.91(0.56-1.49) | 0.721 |  | 0.78(0.39-1.59) | 0.498 |
| LDL cholesterol(mmol/L) |  |  |  |  |  |  |  |  |  |  |  |  |  |  |
| Quartile 1(<1.47) | 52 | 44(84.62) | 172.42 | Reference |  |  | Reference |  |  | Reference |  |  | Reference |  |
| Quartile 2(1.47-1.91) | 54 | 47(87.04) | 130.17 | 1.49(0.98-2.28) | 0.065 |  | 1.50(0.98-2.30) | 0.062 |  | 1.35(0.87-2.10) | 0.176 |  | 1.10(0.62-1.93) | 0.752 |
| Quartile 3(1.91-2.47) | 55 | 45(81.82) | 204.08 | 0.85(0.55-1.30) | 0.443 |  | 0.86(0.55-1.32) | 0.487 |  | 0.79(0.50-1.24) | 0.301 |  | 0.50(0.24-1.07) | 0.075 |
| Quartile 4(≥2.47) | 67 | 55(82.09) | 238.25 | 0.89(0.59-1.35) | 0.594 |  | 0.89(0.59-1.35) | 0.590 |  | 0.77(0.49-1.21) | 0.260 |  | 0.43(0.17-1.10) | 0.079 |
| Triglyceride(mmol/L) |  |  |  |  |  |  |  |  |  |  |  |  |  |  |
| Quartile 1(<0.84) | 57 | 47(82.46) | 169.50 | Reference |  |  | Reference |  |  | Reference |  |  | Reference |  |
| Quartile 2(0.84-1.08) | 66 | 54(81.82) | 225.33 | 0.76(0.51-1.15) | 0.196 |  | 0.77(0.51-1.18) | 0.230 |  | 0.70(0.45-1.09) | 0.112 |  | 0.78(0.49-1.25) | 0.305 |
| Quartile 3(1.08-1.66) | 66 | 60(90.91) | 205.67 | 0.94(0.63-1.39) | 0.755 |  | 0.95(0.64-1.41) | 0.803 |  | 0.80(0.52-1.24) | 0.327 |  | 0.87(0.54-1.43) | 0.591 |
| Quartile 4(≥1.66) | 39 | 30(76.92) | 144.42 | 0.70(0.43-1.14) | 0.151 |  | 0.72(0.44-1.17) | 0.182 |  | 0.58(0.34-1.01) | 0.055 |  | 0.60(0.31-1.17) | 0.134 |
| HDL, high density lipoprotein; LDL, low density lipoprotein; SBP, systolic blood pressure; DBP, diastolic blood pressure; BMI, body mass index. Model 1: adjusted for age, sex, category of residence, marital status, economic income, smoke and drink; Model 2: further adjusted for SBP, DBP and BMI based on model 1; Model 3: further adjusted for blood urea nitrogen, plasma creatine, urea acid and plasma glucose based on model 2; Model 4: further adjusted for total cholesterol, HDL cholesterol, LDL cholesterol and triglyceride based on model 3. | | | | | | | | | | | | | | |
